# Quantifying lumbar sagittal plane kinematics using a wrist-worn inertial measurement unit

**DOI:** 10.3389/fspor.2024.1381020

**Published:** 2024-05-14

**Authors:** Bernard X. W. Liew, Oscar Crisafulli, David W. Evans

**Affiliations:** ^1^ School of Sport, Rehabilitation and Exercise Sciences, University of Essex, Colchester, United Kingdom; ^2^ Criams-Sport Medicine Centre Voghera, University of Pavia, Pavia, Italy; ^3^ School of Sport, Exercise and Rehabilitation Sciences, College of Life and Environmental Sciences, University of Birmingham, Birmingham, United Kingdom

**Keywords:** spine, mobility, wearable sensor, biomechanics, range of motion

## Abstract

Wearable sensors like inertial measurement units (IMUs), and those available as smartphone or smartwatch applications, are increasingly used to quantify lumbar mobility. Currently, wearable sensors have to be placed on the back to measure lumbar mobility, meaning it cannot be used in unsupervised environments. This study aims to compare lumbar sagittal plane angles quantified from a wrist-worn against that of a lumbar-worn sensor. Twenty healthy participants were recruited. An IMU was placed on the right wrist and the L3 spinal level. Participants had to position their right forearm on their abdomen, parallel to the floor. Three sets of three consecutive repetitions of flexion, and extension were formed. Linear mixed models were performed to quantify the effect of region (lumbar vs. wrist) on six outcomes [minimum, maximum, range of motion (ROM) of flexion and extension]. Only flexion ROM was significantly different between the wrist and lumbar sensors, with a mean of 4.54° (95% CI = 1.82°–7.27°). Across all outcomes, the maximal difference between a wrist-worn and lumbar-worn sensor was <8°. A wrist-worn IMU sensor could be used to measure gross lumbar sagittal plane mobility in place of a lumbar-worn IMU. This may be useful for remote monitoring during rehabilitation.

## 1 Introduction

Lumbar mobility is thought to be important for understanding the risk of low back pain (LBP) (1), its recovery, and persistence (2, 3). The construct of lumbar mobility is also tightly integrated within the philosophy of many treatment approaches, such as the movement system impairment approach (4), cognitive functional therapy (CFT) approach (5), and the mechanical diagnosis and therapy, McKenzie, approach (6).

The gold-standard method of measuring lumbar mobility is either video fluoroscopy (7) or bone-pin studies (8). Neither of these techniques can be adopted routinely clinically or in research, due to issues surrounding potential harm from radiation exposure, invasiveness, and lack of equipment. Lumbar mobility can also be assessed using optical motion cameras, with surface-mounted reflective markers (9), and inertial measurement units (IMUs) (10), with studies reporting excellent reliability [e.g., test-retest intraclass correlation coefficient >0.85 (11, 12)]. With significant recent advancements in smartphone sensor technology and software applications (apps), coupled with near-ubiquity of smartphone ownership, smartphones have emerged as a viable clinical solution for the measurement of lumbar mobility (13).

When using wearable sensors such as IMUs or those embedded within smartphones, decisions have to be made on the optimal location of sensor placement (10, 14). A previous study reported that optimal position for the placement of an IMU was 25% of the distance from the midpoint of the posterior superior iliac spines to C7, when compared to the reference standard of optical motion capture (14). When using a smartphone app (TiltMeter^©^) for measuring lumbar mobility, another study required a clinician to position the smartphone on T12 of the subject's spine (15). A requirement for positioning sensors on the lumbar spine limits mobility assessments to a supervised environment, with assistance from trained personnel. This precludes the ability for monitoring lumbar mobility remotely, in free-living unsupervised environments.

**Figure 1:**
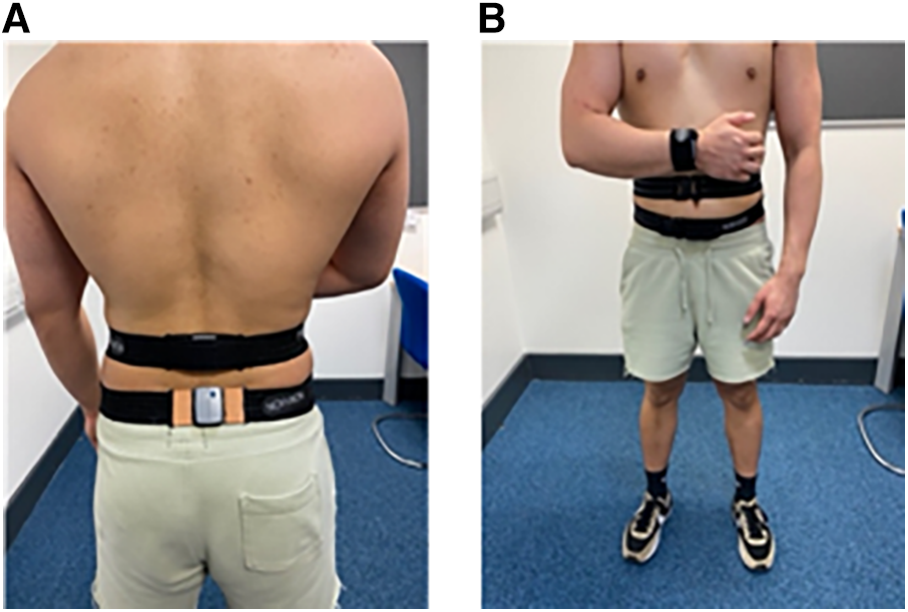
(**A**) A posterior view showing the position of the lumbar and pelvis sensors, (**B**) an anterior view showing the position of the wrist sensor.

A potential solution to these limitations for lumbar sensor placement is to use sensors within wrist-worn smartwatches. For example, the Apple Watch Ultra (Apple Computers Inc., Cupertino, CA, USA) has native apps that utilise the in-built inclinometer (e.g., the “Compass” app) (16). Guo and colleagues demonstrated that the inclinometer on smartwatches can be used to detect and translate various hand gestures into input signals (17). To date, sensors within smartwatches have been used primarily for physical activity-monitoring (18). However, it is possible that by positioning the forearm on the abdomen parallel to the floor (e.g., Figure 1), a wrist-worn IMU could approximate the quantification of lumbar mobility of a lumbar-worn IMU. This present study aims to compare lumbar sagittal plane angles quantified from a wrist-worn sensor against that of a lumbar-worn sensor. We hypothesise that the lumbar sagittal plane angles will be similar when quantified using a wrist- vs. a lumbar-worn IMU sensor.

## 2 Materials and methods

### 2.1 Experiment design and participants

This was a cross-sectional study design, in which data collection took place between November 2022 to February 2023 at the University of Essex, UK. Participants were recruited by word of mouth, printed advertisements, and using social media platforms (e.g., Instagram). Healthy participants were eligible for the present study if they met the following inclusion criteria: (1) aged between 18 and 40 years, (2) free from any restrictions in lumbar spinal mobility, and (3) currently free from symptoms emanating from the lumbar region, as self-reported. The study was approved by the University of Essex Ethics Committee (ETH2223-0474). All participants provided signed informed consent prior to study enrolment.

### 2.2 Sample size

Sample size was calculated using the *pwr* package (v1.3-0) in R software (v4.3.0). A previous study reported a correlation magnitude of 0.6 in the lumbar motion angles when comparing a wearable stretch sensor to optical motion cameras (19). To detect a moderate correlation of 0.6 between the lumbar flexion angles measured using the two methods, at a power of 0.8 and an alpha of 0.05, 16 participants were required. 18 adult participants (9 males, 9 females; mean [standard deviation] age 20.8 [1.6] years, body mass 65.8 [12.4], and height 1.7 [0.09] m) who met the eligibility criteria were recruited.

### 2.3 Instruments

Three IMUs sampling at 200 Hz (Noraxon, USA) were attached to the participant via adjustable straps supplied by the manufacturer (Figure 1). One sensor was positioned on the spinous process of the third lumbar (L3) vertebra, which was identified through manual palpation. Another sensor was placed on the dorsal surface of the distal right forearm. The third sensor was placed on the posterior surface of the pelvis. The pelvic sensor was required to enable the quantification of segment angles relative to a static standing calibration posture (Figure 1). The pelvic sensor was not used for any other calculations.

### 2.4 Data collection

Self-reported age (years), body mass (kg), height (m), and gender were collected via online questionnaire (Qualtrics). For all procedures, participants were instructed to stand with the feet shoulder-width apart, and pointing forwards, as judged by the assessor. Participants performed three sets of three consecutive repetitions of full spinal flexion movements (i.e., nine repetitions in total). Each set was interspersed by a static rest in the standing position of one minute duration. Using a metronome (Pro Metronome, free version) on a tablet computer (iPad, Apple Computers, Cupertino, CA) set to 20 beats per minute (20), all spinal movements occurred at a fixed pace with each half-cycle of movement matching the metronome frequency (e.g., “beat” neutral, “beat” maximally flexed, “beat” neutral, etc.). The IMU sensor was calibrated prior to the start of each set with participants stood in an upright position. Participants were allowed to familiarise themselves with the task prior to data collection, to ensure syncing with the movement cadence.

### 2.5 Data processing

All processing was undertaken in Noraxon MyoResearch software (MR3 3.18.98). All signals were not filtered. Two events were visually identified from the angle signal from the lumbar IMU, for each flexion or extension repetition: “start” where the movement began, and “end” where the participant returned to the upright position. The minimum, maximum, and RoM (maximum-minimum) angles for each repletion were automatically calculated by MyoResearch for each repetition and provided as an average value for each set.

### 2.6 Statistical inference

All statistical analyses were performed using R (version 4.2.2), with statistical significance defined by *P*-value <0.05. There were six dependent variables, the minimum, maximum, and RoM for both flexion and extension; the independent variable is the region with two levels, lumbar and wrist. A linear mixed model was used to compare the effects of region on each of the six dependent variables, with a subject-specific random intercept. Assumption testing of normality and equivalence of variance was performed by residual diagnostic plots.

## 3 Results

**Figure 2:**
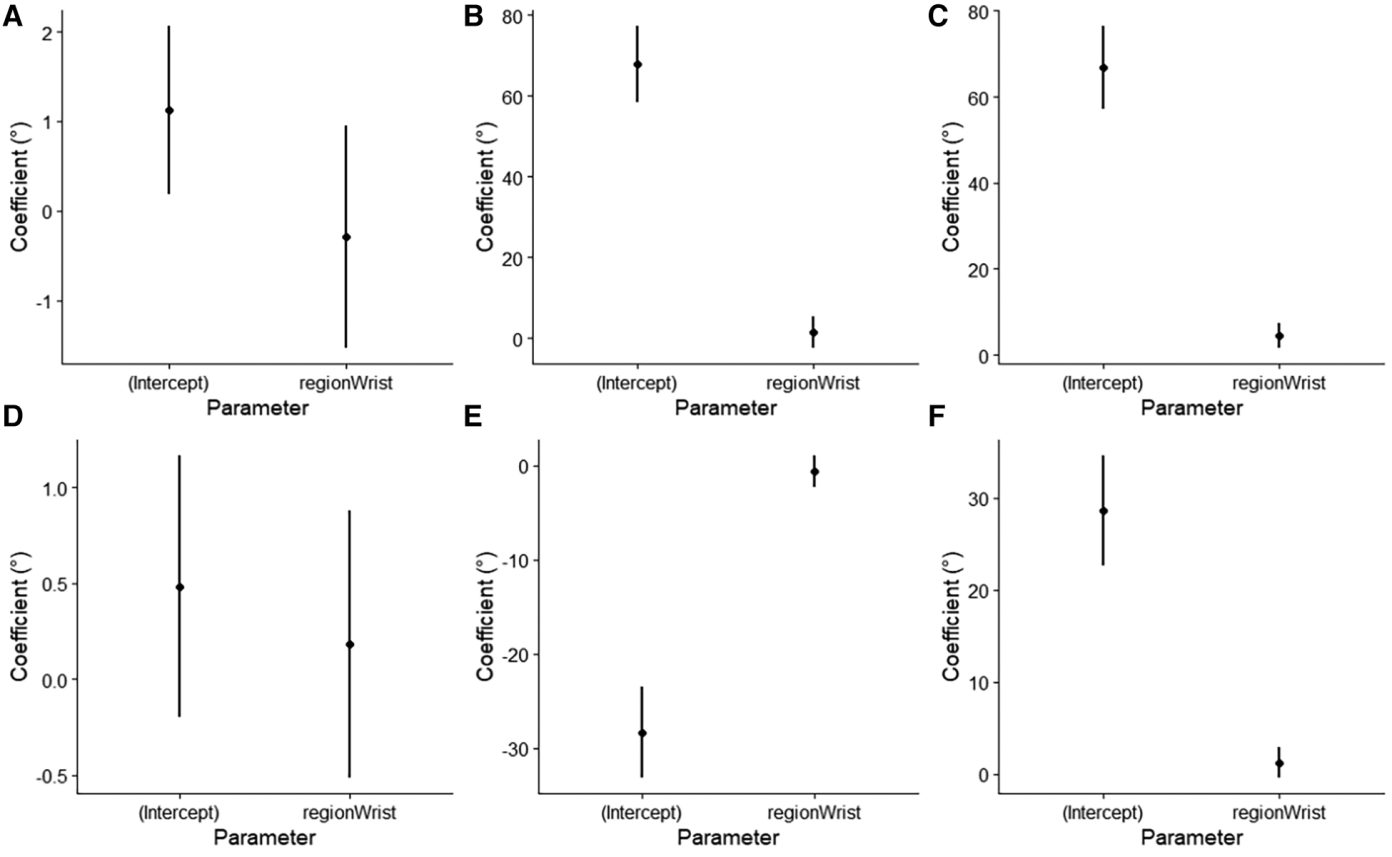
Beta coefficients with 95% confidence interval, of the linear mixed models for (**A**) minimum lumbar flexion angle, (**B**) maximum lumbar flexion angle, (**C**) range of motion of lumbar flexion, (**D**) minimum lumbar extension angle, (**E**) maximum lumbar extension angle, and (**F**) range of motion of lumbar extension.

For the lumbar flexion angle, the minimum angle was not significantly different between the wrist and lumbar sensors, with a mean of −0.29° (95% CI = −1.53°–0.95°, *t* = −0.47, *p* = 0.642). The maximum angle was not significantly different between the wrist and lumbar sensors, with a mean of 1.51° (95% CI = −2.35°–5.36°, *t* = 0.78, *p* = 0.440). The RoM was significantly different between the wrist and lumbar sensors, with a mean of 4.52° (95% CI = 1.59°–7.44°, *t* = 3.07, *p* = 0.003) (Figure 2A-C).

For the lumbar extension angle, the minimum angle was not significantly different between the wrist and lumbar sensors, with a mean of 0.18° (95% CI = −0.55°–0.88°, *t* = 0.52, *p* = 0.605). The maximum angle was not significantly different between the wrist and lumbar sensors, with a mean of −0.66° (95% CI = −2.32°–1.01°, *t* = −0.79, *p* = 0.434). The RoM was not significantly different between the wrist and lumbar sensors, with a mean of 1.22° (95% CI = −0.40°–2.84°, *t* = 1.50, *p* = 0.137) (Figure 2D-E).

## 4 Discussion

Lumbar mobility is thought to be an important contributor to the management of LBP. However, current clinical methods for measuring lumbar mobility require another person to position the IMU sensor on the lumbar spine, which precludes self-monitoring in free-living, unsupervised environments. This study compared lumbar segment flexion and extension angles using a wrist-worn IMU sensor compared to one worn over the lumbar spine. The findings from this study partially confirm the hypothesis as only one out of six tested variables (flexion RoM) displayed a significant difference between the two IMU positions.

Even though one out of six variables investigated was statistically significant between the wrist- and lumbar-worn sensors, the differences may not always be clinically significant. The largest difference observed in the present study was on average <5°, with an upper 95% CI limit of 8°. One study reported that four different LBP subgroups have trunk segmental flexion angles, measured using a single T12 IMU, of 111°, 97°, 89°, and 77° (21). Another study reported a difference of 23° in maximal lumbar flexion RoM between individuals with and without LBP when putting on a sock (22). Clinically, a recovery of LBP has also been associated with a mean change of lumbar sagittal plane RoM by 6 to 10° (23). A case-series study reported a large change in trunk segmental flexion angle (T12 IMU) by −25° was associated with a reduction in LBP intensity, but day-to-day fluctuations in mobility can be <5° (16). This suggests that a wrist-worn sensor would not be suitable for measuring small fluctuations in lumbar mobility.

No studies to our knowledge have compared a wrist-worn IMU against a lumbar-worm IMU for measuring lumbar mobility. However, the differences in angles between regions obtained in the present study were within the differences in angles obtained in a previous study when comparing different spinal positions for an IMU placement (14). When compared against optoelectric motion capture, one study reported that different lumbar sensor placements resulted in a root mean squared error varying from a maximum of −12.5° to a minimum of −5° (14). This suggests that the difference in lumbar segmental flexion angle measured using a single IMU between different spinal positions is −7.5° (14).

This study is not without limitations. First, our participants had a very narrow and healthy BMI range. Given that the wrist-worn method requires placement of the forearm on the abdomen, the accuracy of this method may be affected by people with different anthropometric characteristics. Second, the present study investigated the differences between sensors at one movement speed. It is interesting to speculate if the results found presently will generalise to faster movement speeds. A previous study reported that the lumbar flexion angle measured using an IMU of the same spinal bending task did not change when increasing movement speed from 20 beats/min to 50/beats per min (24). However, because the forearm has to be positioned on the abdomen (Figure 1), higher spinal movement speeds may pose a greater challenge to maintaining the forearm in the calibrated position, thus potentially compromising the accuracy of the wrist-worn method.

In conclusion, a wrist-worn IMU sensor could be used to measure gross lumbar sagittal plane mobility in place of a lumbar-worn IMU. This may be especially useful for remote clinical monitoring during rehabilitation when an external person is not available to place a sensor on the lower back.

## Data Availability

The raw data supporting the conclusions of this article will be made available by the authors, without undue reservation.
